# Canine perspective-taking

**DOI:** 10.1007/s10071-022-01736-z

**Published:** 2023-01-11

**Authors:** Ludwig Huber, Lucrezia Lonardo

**Affiliations:** Comparative Cognition, Messerli Research Institute, University of Veterinary Medicine Vienna, Medical University of Vienna, University of Vienna, Veterinaerplatz 1, 1210 Vienna, Austria

**Keywords:** Dogs, Theory of mind, Mindreading, Behaviour reading, Perspective-taking, Social cognition, Cognitive evolution, Comparative cognition, Guesser-knower task, False-belief task

## Abstract

An important question in the study of canine cognition is how dogs understand humans, given that they show impressive abilities for interacting and communicating with us. In this review, we describe and discuss studies that have investigated dogs’ perspective-taking abilities. There is solid evidence that dogs are not only sensitive to the gaze of others, but also their attention. We specifically address the question whether dogs have the ability to take the perspective of others and thus come to understand what others can or cannot perceive. From the latter, they may then infer what others know and use this representation to anticipate what others do next. Still, dogs might simply rely on directly observable cues and on what they themselves can perceive when they assess what others can perceive. And instead of making inferences from representations of others' mental states, they may have just learned that certain behaviours of ours lead to certain outcomes. However, recent research seems to challenge this low-level explanation. Dogs have solved several perspective-taking tasks instantly and reliably across a large number of variations, including geometrical gaze-following, stealing in the dark, concealing information from others, and Guesser/Knower differentiation. In the latter studies, dogs' choices between two human informants were strongly influenced by cues related to the humans’ visual access to the food, even when the two informants behaved identically. And finally, we review a recent study that found dogs reacting differently to misleading suggestions of human informants that have either a true or false belief about the location of food. We discuss this surprising result in terms of the comprehension of reality-incongruent mental states, which is considered as a hallmark of Theory of Mind acquisition in human development. Especially on the basis of the latter findings, we conclude that pet dogs might be sensitive to what others see, know, intend, and believe. Therefore, this ability seems to have evolved not just in the corvid and primate lineages, but also in dogs.

## Introduction

In this paper, we will review studies about the perspective-taking ability of domesticated dogs. The main aim here is to review recent data that suggest dogs as a species have this capacity, rather than discuss the conditions under which this may occur. We focus on pet dogs and on human behaviour and communication, because most studies have investigated how pet dogs perceive humans rather than conspecifics, and there are many more studies on pet dogs than other canine populations. An additional reason is that pet dogs live in close relationship with humans—only a small subset with one or few conspecifics—have often been raised by humans, are fed by humans, and sometimes establish very strong, attachment-like bonds with humans (Gácsi et al. 2013; Karl et al. 2020; Topál and Gácsi 2012). We do not know if this is the only environment where advanced perspective-taking abilities develop in dogs, but we consider it as a very favourable one.

Overall, the ability to represent the *mental states* of others, such as knowledge, beliefs, intentions, desires, and goals, has been categorized under the umbrella term “Theory of mind” (Premack and Woodruff 1978; Westra and Carruthers 2018). It is widely agreed that such mind-reading (or mentalizing, mental state attribution) has profound impacts on the ways in which we interact with others. Learning about another individual’s mental states would enable us to interpret, predict, and manipulate the behaviour of others (Lewis and Krupenye, in press). Theory of mind is, however, not a unitary cognitive process (Allen 1998), but can be decomposed into unique socio-cognitive skills that have divergent evolutionary and developmental roots (e.g., Baillargeon et al. 2016; Emery and Clayton 2016; Gómez 1996; Martin and Santos 2016; Wellman 2018; Whiten 2013). We can distinguish several simpler processes of behaviour reading and perspective-taking from the mind-reading abilities that provide an understanding of others’ cognitive states such as false beliefs (see Table 1 for possible components of Theory of Mind).

**Table 1:** Cognitive processes of behaviour reading and perspective taking

Phenomenon	Functional description
Following others’ gaze	Detecting others’ gaze direction and following it
Following others’ gaze into distant space	Actively using of others’ gaze cues; considered as a crucial step towards an understanding of mental states like attention and intention
Geometrical gaze-following	Tracking others’ gaze direction geometrically behind visual barriers
Sensitivity to others’ attention	Recognising and reacting appropriately to different cues of visual attention; having some understanding of others’ visual field and make use of this information in a functional way both in cooperative (e.g., begging, obeying a command) and competitive contexts (e.g., stealing)
Level 1 perspective-taking	Discerning what another individual can and cannot perceive from her own point of view
Egocentric perspective-taking	Relying on what one can perceive when assessing what others can perceive
Altercentric perspective-taking	Using the other's perspective in an altercentric manner; mentally reposition oneself to imagine what others have perceived or could potentially perceive
Knowledge attribution	Understanding that seeing in the past leads to knowing in the present; inferring from the concept of seeing what others know and do next
Level 2 perspective-taking	Forming a mental representation of the mental states of the other; understanding not only *what* can and cannot be perceived from a certain point of view but also *how* a given situation is perceived or represented; understanding that the same situation might appear differently from another’s perspective

While, for humans, the main question is when and under what circumstances these "higher" mind-reading processes emerge during development (for reviews, see Baillargeon et al. 2016; Grosse Wiesmann et al. 2017), the corresponding research on non-human animals has been primarily motivated by the question whether such skills exist at all outside the genus Homo (Buckner 2014; Heyes 2015; Lurz 2011). Since the seminal paper by Premack and Woodruff (1978), it is mainly great apes that have been tested in such tasks (for reviews, see Call and Tomasello 2008; Krupenye and Call 2019; Lewis and Krupenye, in press). However, other primates (Hayashi et al. 2020; Horschler et al. 2020), other mammals, and even birds—especially corvids (see, for instance, Bugnyar et al. 2016; Emery and Clayton 2004, 2016)—have been added to make broader phylogenetic comparisons. The latter taxa have proved especially useful for highlighting cases of convergent evolution and socioecological pressures of mind-reading abilities.

A particularly interesting and valuable model in this respect is the domestic dog. Researchers have proposed convergent evolution with humans through the process of domestication, with selection favouring social skills for cooperation in dogs that were also important in the evolution of our species (Hare and Tomasello 2005; MacLean et al. 2017; Topál et al. 2009a, b). Indeed, over the past few decades, dog researchers who have investigated the relationship between dogs and humans have found that dogs have evolved to exhibit socio-cognitive abilities that are not found in other species, including their closest living relatives, the wolves (for recent studies, see Bray et al. 2021; Salomons et al. 2021). This has led researchers to suggest that dogs are born to be ‘human whisperers’ (Kaminski 2021). But apart from their ability to read communicative human gestures such as pointing (Kaminski and Nitzschner 2013; Krause et al. 2018; Miklósi et al. 1998), can dogs make inferences about humans’ mental states?

The mundane alternative would be that they love us (Berns 2014; Wynne 2019) and therefore are very interested and sensitive to what we are doing. By constantly monitoring humans, especially their caregiver(s), they not only perceive what they are currently doing, but infer what they are interested in and are doing next. They spontaneously focus attention on informative objects, such as eyes (Somppi et al. 2012, 2014), discriminate our emotions (Albuquerque et al. 2016; Müller et al. 2015), and are sensitive to human attentional states (Call et al. 2003; Mongillo et al. 2010; Schwab and Huber 2006; Virányi et al. 2004) from puppy age on (Gácsi et al. 2005). Some have therefore argued that success in perspective-taking tasks might be based on associations formed during the experiment or in earlier life or simply on reading others’ behaviour and acting on the basis of that information (Penn and Povinelli 2007; Roberts and Macpherson 2011; Udell et al. 2011). This hypothesis would find empirical support from at least two facts: (1) that grey wolves are also sensitive to human attentional state under some conditions and (2) that dogs do not display an undifferentiated sensitivity to all visual cues of attentional state (Udell et al. 2011). Furthermore, dogs are more sensitive to stimuli encountered in their home environment and show great inter-individual differences in performance due to different life histories and experience with humans.

To evaluate the various hypotheses systematically, we will start this review by reporting findings that show how well dogs interpret humans’ attentive states to behave appropriately (see Table 2 for overview of the reviewed publications). We then describe and discuss the most recent experiments that suggest some kind of sensitivity to humans’ perspectives, especially in the visual, but also in the auditory domain (Table 2). Then, we discuss reports of knowledge attribution by applying a concept of seeing (Table 3). And finally, we provide first evidence of the comprehension of reality-incongruent mental states, or false beliefs, of others (Table 3), which is considered as a hallmark of ToM acquisition in human development (Wimmer and Perner 1983). Especially, on the basis of the latter findings, we come to the conclusion that pet dogs might be sensitive to what others see, know, intend and believe, and therefore, this ability seems to have evolved outside the corvid and primate lineage.

**Table 2:** Details of experiments investigating dogs' sensitivity to others' gaze and to others' attention

Authors	Year	Title	Finding
Sensitivity to others' gaze			
Call et al.	2003	Domestic dogs (Canis familiaris) are sensitive to the attentional state of humans	Dogs are sensitive to human attentional states
Virányi et al.	2004	Dogs respond appropriately to cues of humans’ attentional focus	Dogs obey a command more promptly if the human is facing them
Gacsi et al.	2005	Species-specific differences and similarities in the behaviour of hand-raised dog and wolf pups in social situations with humans	Dogs are sensitive to human attentional states
Schwab and Huber	2006	Obey or not obey? Dogs (Canis familiaris) behave differently in response to attentional states of their owners	Dogs are sensitive to human attentional states
Mongillo et al.	2010	Selective attention to humans in companion dogs, Canis familiaris	Dogs are sensitive to human attentional states
Met et al.	2014	Gaze-following behind barriers in domestic dogs	Dogs are capable of following human's gaze geometrically
Wallis et al.	2015	Training for eye contact modulates gaze-following in dogs	Dogs can follow human gaze into distant space
Duranton et al.	2017	Do pet dogs (Canis familiaris) follow ostensive and non-ostensive human gaze to distant space and to objects?	Dogs follow human gaze even without ostensive cues in an object-choice task
Sensitivity to others' attention			
Hare et al.	1998	Communication of Food Location Between Human and Dog (Canis Familiaris)	Dogs can take into account humans’ field of view when communicating and interacting with us
Cooper et al.	2003	Clever hounds: social cognition in the domestic dog (Canis familiaris)	Dogs are more likely to beg or seek interaction from a person who can see the dog or the reward being requested than someone who cannot
Gacsi et al.	2004	Are readers of our face readers of our minds? Dogs (Canis familiaris) show situation-dependent recognition of human's attention	Dogs discriminate between attentive and inattentive humans
Udell et al.	2011	Can your dog read your mind? Understanding the causes of canine perspective-taking	Dogs are more likely to beg or seek interaction from a person who can see the dog or the reward being requested than someone who cannot
Savalli et al.	2013	Are dogs sensitive to the human’s visual perspective and signs of attention when using a keyboard with arbitrary symbols to communicate?	Dogs can take into account humans’ field of view when communicating and interacting with us
Marshall-Pescini et al.	2013	Gaze alternation in dogs and toddlers in an unsolvable task: evidence of an audience effect	Dogs show susceptibility to an audience effect depending on a human recipient’s attentional state
Savalli et al	2014	Are Dogs Able to Communicate with Their Owners about a Desirable Food in a Referential and Intentional Way?	Dogs show susceptibility to an audience effect depending on a human recipient’s attentional state
Savalli et al.	2016	Eye Contact Is Crucial for Referential Communication in Pet Dogs	Dogs take into account a human’s visual attentional state when communicating
Kaminski et al.	2017	Human attention affects facial expressions in domestic dogs	Dogs can take into account humans’ field of view when communicating with us
Bryant et al.	2018	Roles for referential focus in effective and efficient canine signaling: Do pet and working dogs differ?	Dogs obey a command more promptly if the human is facing them
Kiss and Topál	2019	How do dogs monitor the human’s attentional state after challenged by the presence of forbidden food?	Dogs are susceptible to audience effect, but there are differences between individuals in terms of the readiness to conform to the human expectations
Kiss et al.	2020	Behavioural and neurophysiological correlates of dogs’ individual sensitivities to being observed by their owners while performing a repetitive fetching task	Dogs show susceptibility to an audience effect depending on a human recipient’s attentional state

**Table 3:** Details of experiments investigating visual perspective-taking, knowledge attribution, and sensitivity to others’ beliefs in dogs

Authors	Year	Title	Finding	Results^a^	Sample size	Exp. design	*N* test trials
Visual perspective-taking							
Bräuer et al.	2004	Visual perspective-taking in dogs (Canis familiaris) in the presence of barriers	Dogs are sensitive to the properties of barriers as blocking devices for visual access in humans; dogs seem to use an allocentric perspective rather than egocentric perspective to gauge the visual access of others under various conditions; but no evidence suggesting that dogs have access to what they themselves have seen	Exp. 1 = average percentage of trials in which the dogs ate the forbidden treat when this was hidden behind a barrier: 80%; when the barrier did not hide the treat from the experimenter: 56%. Percentages inferred from Fig. 2. Exp. 2 = average percentage of trials in which the dogs ate the forbidden treat when this was hidden behind a large barrier concealing both their approach and their food intake: 76%; when only their approach was concealed by a large barrier with a window: 69%; when only the food taking was concealed by a small barrier: 63%. Percentages inferred from Fig. 4	10 (Exp. 1)—17 (Exp. 2)	Within-subject	8 per condition
Kaminski et al.	2009	Domestic dogs are sensitive to a human's perspective	Dogs are able to take into account the human’s perspective rather than extrapolating from their own perspective	Exp. 1 = average percentage of trials in which dogs chose the toy behind the transparent barrier when this was the only toy visible to the experimenter: approx. 73%; when none of the toys was visible to the experimenter: approx. 62%; when both toys were visible to the experimenter: approx. 52%. Percentages inferred from Fig. 2	47	Between-subjects	8
Kundey et al.	2010	Domesticated dogs (Canis familiaris) react to what others can and cannot hear	Dogs steal food from a silent but not from a noisy container	Dogs that approached the silent container when the experimenter was looking at them: 40%; when the experimenter was not looking at them: 100%	20	Between-subjects	1
Kaminski et al.	2013	Dogs steal in the dark	Dogs take into account the human’s visual access to food while making their decision to steal it; they steal more food when it is dark compared to when it is light; and they hesitate longer to steal food when the food is illuminated, but only when an experimenter is present in the room (irrespectively of whether the experimenter is illuminated or not)	Exp. 1 = average percentage of trials in which the dogs stole the food when the experimenter but not the food was illuminated: 66%; when the food but not the experimenter was illuminated: 60%; when both were illuminated: 41%; when neither was illuminated: 83%	28 (Exp.1)—12 (Exp.2)	Within-subject	16 (Exp.1)—8 (Exp.2)
Bräuer et al.	2013	Domestic dogs conceal auditory but not visual information from others	Dogs steal food from a silent but not from a noisy container; but fail to inhibit stealing when they be seen; dogs failed to overcome an egocentric perspective to decide what the human can potentially see or not	Exp. 1 (concealing visual cues of approach) = dogs that chose to approach the food through the opaque tunnel when the experimenter remained visible after forbidding to take the food: 52%; when the experimenter left the room after forbidding to take the food: 43%; when the experimenter encouraged the dogs to take the food: 40%. Exp.2 (concealing auditory cues of approach) = dogs that chose to approach the food through the silent tunnel when the experimenter remained visible after forbidding to take the food: 87%; when the experimenter left the room after forbidding to take the food: 53%; when the experimenter encouraged the dogs to take the food: 44%	67	Between-subjects	1
MacLean et al.	2014	Dogs (Canis familiaris) account for body orientation but not visual barriers when responding to pointing gestures	Dogs are skilled both at following human gestures, and exploiting information about others’ visual perspectives, they may not integrate these skills in the manner characteristic of human children	Exp. 1: Dogs were more likely to follow a pointing gesture from an individual who faced (62%) them compared with one with his back turned (55%). Exp. 2 = average percentage of trials in which dogs chose the container closer to the experimenter when this was also the only one visible to the experimenter: 90%; when it was not the only one visible to the experimenter: 83%. Exp. 3 = average percentage of trials in which dogs chose the container closer to the experimenter when this was not visible to the experimenter: 96%; when both containers were visible to the experimenter (no barrier): 93%. Exp. 4 = average percentage of trials in which dogs chose the container behind the transparent barrier when this was the only treat visible to the experimenter: 48%; when the experimenter could see both containers due to the orientation of the barriers: 49%	30 (Exp. 2)—20 (Exp. 3)—30 (Exp. 4)	Within-subject	8 (ambiguous condition)
Heberlein et al.	2017	Dogs’ (Canis familiaris) attention to human perception: Influence of breed groups and life experiences	Dogs discriminate between opaque and transparent barriers in order to understand which one is blocking the view of a human actor	Exp. 1 = Dogs that ate first the treat not visible to the owner when only one treat was hidden from the owner: 63.7%;	171 (Exp.1)	Within-subject	2
Knowledge attribution							
Cooper et al.	2003	Clever hounds: social cognition in the domestic dog (Canis familiaris)	Knower–Guesser: dogs have convincingly preferred the knower	In the first trial, 93% of the dogs chose the knower. On average, dogs chose the knower on 64% of the trials	15	Within-subject	6
Tópal et al.	2006	Mindreading in a dog: An adaptation of a primate 'mental attribution' study	A dog could differentiate “knowledgeable” and “ignorant” states of its human partner on the basis of whether human participated in object hiding events or not	Trials in which the dog indicated the location of the key to open a box, on average between the conditions in which the owner already knew the location of the key: 18.75%; in the condition in which the owner was ignorant about the location of the key: 75%	1	Within-subject	(8 per condition) 24
Virányi et al.	2006	A non-verbal test of knowledge attribution: a comparative study on dogs and children	Dogs failed to indicate to the owner where a desired but inaccessible toy was hidden in the absence of the owner	On average between sessions and conditions, dogs that indicated the location of the toy when the experimenter was aware of it: 70.75%; when the experimenter was ignorant of it: 88.75%; dogs that indicated the location of the stick when the experimenter was aware of it: 13.5%; when the experimenter was ignorant of it: 6.75%	11	Within-subject	8 (2 in each condition)
Kaminski et al.	2009	Domestic dogs are sensitive to a human's perspective	Dogs failed to bring on command the toy the experimenter had previously seen and therefore knew of as opposed to a toy the experimenter was not aware of	Dogs retrieved the toy whose hiding had been witnessed by the experimenter in 45% of the trials	8	Within-subject	8
Maginnity and Grace	2014	Visual perspective-taking by dogs (Canis familiaris) in a Guesser–Knower task: evidence for a canine theory of mind?	When informants had differing perceptual access to the baiting, dogs preferred the location indicated by the Knower from the start of testing, even when baiting was done by a third experimenter	Exp. 1 = trials in which the dogs chose the knower in the Guesser Absent condition: 73%; in the Guesser Present condition: 58%. In the first trial, dogs that chose the knower in the Guesser Absent condition: 81.3%; in the Guesser Present condition: 50%. Exp.2 = trials in which the dogs chose the knower when the knower covered her cheeks with her hands and the guesser covered her eyes: 64%. Exp.3 = trials in which the dogs chose the knower when the knower looked at the baiting and the guesser looked at the ceiling: 62%. Exp.4 = trials in which the dogs chose the knower when the knower and guesser had equal knowledge of the location of the food: 53%; when they equally ignorant of the location of food: 47%; trials in which dogs chose the baited container when neither of the informants pointed at a cup (olfactory cues control): 4.2%	16 (Exp. 1 and 2)—12 (Exp. 3 and 4)	Within-subject	24 per condition
Catala et al.	2017	Dogs demonstrate perspective-taking based on geometrical gaze-following in a Guesser–Knower task	Dogs preferred to follow the pointing of a human who witnessed a food hiding event over a human who did not; they even did so (from the first trial on) when the two informants showed identical looking behaviour, but differed in their position in the room, so that one had the opportunity to see where the food was hidden by a third person, the other not	Trials in which the dogs chose the Knower in the Guesser Absent condition: 72.3%; in the Guesser Looking Away condition: 61.7%; in the Guesser Present condition: 56.2%. In the first trial, dogs that chose the Knower in the Guesser Absent condition: 73.3%; in the Guesser Looking Away condition: 81.3%; in the Guesser Present condition: 62.5%	16	Within-subject	24 per condition
Sensitivity to others’ beliefs							
Lonardo et al.	2021	Dogs follow human misleading suggestions more often when the informant has a false belief	On average, dogs chose the suggested container significantly more often in the FB group than in the TB group and hence were sensitive to the experimental manipulation. Terriers were the only group of breeds that behaved like human infants and apes by following the communicator’s suggestion more often in the TB than in the FB group	Dogs that followed the misleading suggestion in the FB group (Exp.1): 48%; in the TB group (Exp.1): 29%; in the Retroactive Interference—Control TB group (Exp. 2): 28%; Border collies that followed the misleading suggestion in the FB group (Exp. 3): 55%; in the TB group: 30%; Terriers that followed the misleading suggestion in the FB group (Exp. 3): 20%; in the TB group: 50%	260	Between-subjects	1

^a^Note, we report the results of only one dependent variable, although some studies measured more than one

## Sensitivity to others' gaze

The most basic level concerns simple detection of others’ gaze direction, particularly the response to being looked at (Ohkita et al. 2016). Gaze detection is widespread among vertebrates and seems to be based on relatively simple mechanisms (Emery 2000). A second level of gaze responsiveness concerns the following of others’ gaze into distant space (Povinelli and Eddy 1996). This active use of others’ gaze cues has been considered a crucial step towards an understanding of mental states like attention and intention (Tomasello et al. 2005). Following human gaze in dogs can be considered a socially facilitated orientation response, which in object-choice tasks is modulated by human-given ostensive cues. Using the traditional test paradigm utilized for human infants (Scaife and Bruner 1975) and by testing a large sample of 145 Border collies, Wallis et al. (2015) provided the first evidence that dogs can follow human gaze into distant space.

A follow-up study in the Clever Dog Lab Vienna not only confirmed the previous findings, but showed that extensive communication (eye contact with repetitive calls and gaze shifts) is necessary to trigger reliable gaze-following into distant space in pet dogs (Duranton et al. 2017). Interestingly, dogs follow human gaze even without ostensive cues in an object-choice task, in which the demonstrator looked in the direction of a potential food container. This result suggests that gaze cues that are congruent with object locations are more effective than gaze cues into open space, much like in human infants (Senju et al. 2008).

Cognitively more sophisticated mechanisms are required for ‘geometrical gaze following’, in which subjects track others’ gaze direction geometrically behind visual barriers (Tomasello et al. 1999). Great apes and several monkey species can do this, and check back with an individual if they cannot pinpoint the target of their gaze (for review, see Lewis and Krupenye, in press). It suggests that subjects appreciate the difference between their own and another’s line of sight and understand that if another’s eyes are directed towards a location behind a barrier, it must alter its own position to see the object of its interest (Povinelli and Eddy 1996). In addition to a handful of non-primate species (Fitch et al. 2010; Schloegl et al. 2008), dogs seem to possess this ability (Met et al. 2014). This ability requires a constant monitoring, i.e., looking at the human and being attentive to what he or she is doing (Emery 2000). Dogs monitor humans, especially their caregiver(s), not only to know what they are currently doing, but also in what they are interested in and therefore are doing next. Evidence for this comes from social referencing tasks, in which dogs used the emotional information provided by an informant about a novel object/stimulus to guide their own future behaviour towards it (Duranton et al. 2016; Merola et al. 2012; Salamon et al. 2020). Some have therefore concluded that the exceptional attentiveness towards humans has an innate component that was probably selected for during domestication (Bräuer 2014).

## Sensitivity to others’ attention

Several studies have now shown that dogs are sensitive to behavioural and environmental cues that are associated with others’ seeing and paying attention: (i) they steal prohibited food more often when humans are distracted, absent, close their eyes, or turn their back to the dog compared to when humans are looking at the dogs intently (Bräuer et al. 2004; Call et al. 2003; Kaminski et al. 2013; Kiss and Topál 2019; Kundey et al. 2012; Schwab and Huber 2006); (ii) they discriminate between attentive and inattentive humans based on orientation of head, body, and visibility of the eyes while playing fetch, obeying commands, and begging for food (Gácsi et al. 2004); (iii) they obey a command more promptly if the human is facing them than when the human orients into distant space, faces a second person (Virányi et al. 2004) or turns her back to them (Bryant et al. 2018; MacLean et al. 2014); (iv) they follow the pointing gestures of a forward-facing experimenter more often than those of an experimenter whose back was turned (MacLean et al. 2014); (v) they are more likely to beg or seek interaction from a person who can see the dog or the reward being requested than someone who cannot (Cooper et al. 2003; Gácsi et al. 2004; Udell et al. 2011); (vi) they take into account humans’ field of view when communicating and interacting with us (Hare et al. 1998; Savalli et al. 2013); (vii) they take into account a human’s visual attentional state when communicating, by increasing communicative behaviours once eye contact is established and when the human attends to them (Kaminski et al. 2017; Savalli et al. 2016); (viii) they show susceptibility to an audience effect across different tasks by modulating their communicative behaviour (gaze alternation) depending on a human recipient’s attentional state (Kiss et al. 2020; Marshall-Pescini et al. 2013; Savalli et al. 2014); and (ix) they are sensitive to and can manipulate conspecifics’ attentional state during play (Horowitz 2009). In sum, dogs recognise and react appropriately to many different cues of visual attention, have some understanding of humans’ visual field, and can make use of this information in a functional way both in cooperative (e.g., begging, obeying a command) and competitive contexts (e.g., stealing).

However, we do not know yet whether their concept of seeing can abstract beyond observable behavioural and environmental cues. Indeed, research on understanding others’ focus of attention has always confronted dogs with tasks in which behavioural and environmental cues differed across conditions (for example, eyes visible in one condition but not in another). Therefore, in order to investigate whether dogs are able to ascribe perception to others, one must not only test whether they recognise the association between certain behavioural or environmental cues and a likely outcome or reaction, but also whether they can correctly infer others’ differential perceptual access in the absence of differential behavioural or environmental cues between conditions. To exemplify, tests are needed in which experimenters behave identically between conditions (e.g., present in the room, with eyes open, facing the dog, orienting towards the scene of interest, etc.…), but in one condition, they can perceive (e.g., see an object of interest or the dog) and in the other they cannot.

## Visual perspective-taking

Central to the question of “mind-reading” is whether non-human animals can form the concept of “seeing”. More specifically, the question is whether non-human animals can attribute the concept “seeing” without relying on behavioural cues, and whether subjects would infer from such a concept, once established, what others know and will do next. Studies with primates (Bräuer et al. 2007; Flombaum and Santos 2005; Hare et al. 2000; Meunier 2017) and corvids (Bugnyar 2011; Bugnyar et al. 2016; Dally et al. 2006; Emery and Clayton 2001) have addressed the ability to decide about the visual access of others to a target object and to use this information either as a basis for choosing an informant to rely on (cooperative tasks), or as a cue for using counter-tactics to secure a desirable object (competitive tasks). The cues of others’ visual access can be more or less obvious, being either directly observable or subtle, but always require a certain degree of perspective-taking. Whether this decision requires the subject to represent, in some form, the mental awareness of others remains an open question (Heyes 2015, 1998; Penn and Povinelli 2007; Povinelli and Vonk 2004). Indeed, in most studies, subjects had to integrate observable features from others’ current or past behaviours, and might have based their decisions solely on their *own* (egocentric) rather than the other’s (altercentric) perspective. Such heuristics do not require representations of others’ mental states, like “knowing” or “seeing”.

Investigations into the question of whether dogs understand what humans can or cannot see started almost 2 decades ago. Going beyond research on dogs’ sensitivity to human attentional states, Bräuer et al. (2004) investigated whether dogs might be capable of Level 1 perspective-taking (Flavell et al., 1981): the ability to understand what others can or cannot see. Consistently with this ability, dogs stole forbidden food more often when the orientation and location of a visual barrier prevented an experimenter from seeing them, compared to when the barrier was present but ineffective in blocking the experimenter’s view (due to its position and orientation). In a subsequent experiment, the authors ruled out the possibility that dogs might have felt more physically protected by the presence of the barrier, which is similar to the explanation that Povinelli and Giambrone (2001) suggested for the behaviour of subordinate and dominant chimpanzees. Dogs reduced the number of stolen treats when they were separated from the experimenter either by a smaller barrier, which did not conceal their approach, or by a large barrier containing a transparent window, which concealed their approach but did not conceal their actual taking of the food. The authors argued that the visibility of the human’s eyes through the transparent window was unlikely to explain this pattern of results. Indeed, the dogs did not hesitate more (i.e., they did not approach the window and then stop moving) once they could potentially see the human eyes through the window, compared to the condition in which the small barrier precluded the visibility of the human eyes when the dogs had already approached the food. But can we exclude the possibility that dogs are just relying on physical features of the environment instead of understanding the human’s visual access to the food (Bräuer 2014)?

Using a fetching task, Kaminski et al. (2009) confirmed dogs’ ability to take into account the human’s perspective rather than extrapolating from their own perspective. When dogs were asked to fetch a toy by an experimenter who could only see a toy behind a transparent barrier, but not another hidden behind an opaque barrier, while dogs themselves could see both from their point of view, they preferentially selected the toy that the experimenter could see. In a second experiment, however, dogs failed to show sensitivity to what the experimenter had witnessed in the past, as they did not prefer to bring the toy the experimenter had witnessed being hidden, rather than a toy whose hiding the experimenter had not witnessed (and was therefore unaware of). Perhaps, dogs only make use of cues present at the time they make a decision, but do not integrate past events to decide what others have seen in the past: see the section “Knowledge attribution” (below) for a more detailed discussion.

A study that used a similar logic, with the dog seeing two objects and the human experimenter just one, failed to clarify the issue (MacLean et al. 2014). Although dogs more often followed the pointing gestures of an experimenter who faced them compared to an experimenter with their back turned, they did not show any sensitivity to the pointer’s perspective when a visual barrier occluded the pointer’s view of one of two containers. However, the experimenter pointed ambiguously into the air in the direction of (but not directly at) two possible locations where food could be hidden, and perhaps these gestures were too vague to be interpreted by the dogs as relevant to finding the food. Similarly confusing might have been the fact that the experimenter baited both containers prior to pointing, and therefore, the dogs might have assumed that the experimenter was aware of the presence of both containers, despite being prevented from seeing one at the moment of pointing. In general, studies investigating dogs' understanding of the human pointing gesture in two-choice tasks have so far not provided unambiguous evidence that it is perceived as a referential signal indicating the exact location where food is hidden rather than the direction to go in (Kaminski and Nitzschner 2013). Moreover, when two possible targets are in the indicated direction, dogs tend to approach the target closer to the experimenter even if the pointing gesture clearly refers to a more distant container (Lakatos et al. 2012).

Evidence for the ability of dogs to discriminate between opaque and transparent barriers to understand which one is blocking the view of a human actor—this time their owner—was provided in a stealing task (Heberlein et al. 2017a, b). The researchers presented dogs with a choice between two pieces of forbidden food, one placed behind an opaque barrier (and hence visible to the dog but not to the owner) and the other behind a transparent barrier (hence visible to both). Overall, dogs stole first (they could then also steal the other one) the treat behind the opaque barrier significantly above chance, thus proving to be sensitive to their owner’s perspective. Interestingly, working style and breed group had an effect on dogs’ choice. While independent workers (such as sight hounds, scent hounds, sledge, and primitive type dogs) were more likely than chance to steal first the treat behind the opaque barrier, cooperative workers (such as shepherds, retrievers, pointers, and guarding dogs) did not show a preference for stealing one of the two treats first.

A certain degree of cleverness at watching what others see when stealing food was shown by dogs in an ingenious study by Kaminski et al. (2013). They found that dogs stole significantly more food in a dark room compared to a situation where a spotlight illuminated the experimenter who forbade them to take the food and another one illuminated the food itself. So far, this is not exciting, as dogs could simply have stolen the food, because they did not see the human and her eyes (Bräuer 2014). However, dogs did not simply take the visibility of the human as a signal to avoid the food, because they waited longer to steal the food when it was illuminated compared to the condition in which the human but not the food was illuminated. In a control experiment in which the experimenter left the room, dogs stole the illuminated food quicker than the non-illuminated food. This suggests that dogs’ faster stealing of non-illuminated food in the previous experiment depended on the presence of the experimenter rather than on a spontaneous preference for stealing food in the dark. A third experiment raised the possibility that the general level of illumination in the room affected the decision to actually steal the food but not the latency to steal, which was again influenced by whether the food was illuminated or not, irrespective of whether the experimenter was illuminated or not. Together, these findings suggest that dogs understand that when the food is illuminated, the human can see them approaching and stealing the food. In summary, dogs seem to use various indirect cues to infer what humans can possibly see or not and to flexibly decide whether to steal, to beg, or to fetch.

## Auditory perspective-taking

Two studies have shown that this surprisingly advanced understanding about others' perspective is not restricted to the visual sense but rather extends into the auditory domain. Again, dogs were forbidden to take food, but this time from both a noisy and a silent, but visually identical, container (Kundey et al. 2010). If they understand that a human who had previously forbidden them to eat food and who is present, but visually inattentive, can still hear their stealing attempt from a noisy container, they should try to get the food from the silent container instead. This is exactly what happened. This preference was only shown when the human was looking away from the dog and food, but not when she was looking in their direction. These results suggest that dogs can take into account what a human can or cannot hear and realise that this information is only relevant when the human is not paying visual attention to the scene. Interestingly, not only pet dogs, but also dogs from an animal shelter performed in this way; therefore, the results generalise to dogs with less human contact.

Additional evidence for auditory perspective-taking came from a study that tested both auditory and visual perspective-taking in a complex stealing context (Bräuer et al. 2013). Dogs could enter an apparatus from the side to steal food by walking on a noisy or silent mat, after a pre-test ensured that they understood they were forbidden from eating the food. Dogs preferred to approach the food from the silent side of the apparatus when the experimenter remained inside the apparatus to listen, but not when she left the apparatus or when she encouraged them to take the food. These contrasts in performance provide another piece of evidence that dogs understand *when* the noise is relevant for being detected by the human. They also suggest that the manipulation of another experiment in this study, in which an opaque and a transparent tunnel was used to test visual perspective-taking ability, might have not been salient enough for the dogs to hide their approach. The idea was to test whether dogs refrain from stealing the food when they see it inside a transparent tunnel, but starting at a position where they could not see a human present. Because dogs failed to insert a paw only into the opaque tunnel, the authors assumed that they did not infer what could possibly be seen by the experimenter. The authors’ conclusion was that, in this set-up, dogs are not able to overcome an egocentric perspective to decide what the human can potentially see or not, in contrast to chimpanzees in a similar task (Melis et al. 2006). In other words, dogs seem to rely on what they themselves can see when they assess what humans can see. However, it is possible that the positioning of the tunnels wholly inside the apparatus, without extending to the outside of the box, made it difficult for dogs to assess their transparency to decide whether to insert their paws and nose to steal the food inside.

In sum, based on convergent evidence from different types of paradigms, one may conclude that dogs are sensitive to several of the behavioural and environmental cues usually associated with seeing or hearing on which they have been tested. However, research prior to 2014 had not been able to rule out the low-level explanation for these abilities, that dogs rely on what they themselves can perceive when they assess what the human can see and hear (Bräuer 2014). Actually, two main questions about dog's perspective-taking ability remained open: (i) whether they can assume the other's point of view irrespective of their own (altercentric perspective-taking) and (ii) whether they can infer from what others *did* see what they later know (knowledge attribution). We turn to these two questions in the following sections.

## Altercentric perspective-taking

The questions whether dogs understand that seeing in the past leads to knowing in the present had been investigated with limited success before 2014 (e.g., Gaunet and Massioui 2014). Two Hungarian teams had used the ‘Ignorant helper’ paradigm, which required the dog to indicate to the owner where a desired but inaccessible toy had been hidden in the absence of the owner, but the results remained inconclusive (Topál et al. 2006; Virányi et al. 2006). And in the fetching task mentioned above (Kaminski et al. 2009), in which the experimenter saw only one object hidden behind a barrier, while the dogs could permanently see two objects, the dogs failed to bring on command the toy the experimenter had previously seen and therefore knew of.

The conclusion that dogs might not be able to attribute knowledge to humans on the basis of what they have seen the humans seeing was challenged when researchers applied the ‘*Guesser–Knower*’ task (Povinelli et al. 1990). This cooperative-communicative task, invented by primatologists, requires the observer to distinguish between knowledgeable and ignorant others by appreciating their differential visual access to a hiding event (Povinelli and Eddy 1996). While one informant (the Knower) either hides the food her/himself or watches someone else hide the food, the other (the Guesser) is out of the room or otherwise cannot see the baiting. Then, the Knower points to the correct container, whereas the Guesser points to an incorrect one. If the subject, who is unaware of the location of food, attributes knowledge of food location to the informant that has seen the baiting, then it should choose the food container that the Knower is pointing to.

While a first study (Cooper et al. 2003) did not convincingly show that dogs solve the task, especially because of a dramatic drop in performance after the first trial, a second study provided substantial evidence in its favour. Maginnity and Grace (2014) showed that dogs’ choices between two human informants were influenced by cues related to the humans’ visual access to the food. In an attempt to determine the cues used by the dogs, the researchers systematically manipulated the human informants’ perceptual access, participation, and knowledge state regarding the food baiting. Importantly, in contrast to the original study with chimpanzees (Povinelli et al. 1990), dogs did not receive discrimination training prior to testing. In four experiments, the dogs unequivocally responded to the differing perceptual access of the human informants to the baiting by preferring the location indicated by the Knower. They not only avoided trusting a human who was absent during the hiding of the food, but also avoided following the suggestion (pointing to a food container) of a human who looked at the ceiling or covered her eyes with her hands during the food hiding process. Importantly, when there was no difference in perceptual access and both informants either knew or did not know the food location, dogs had no preference between the informants. And controls ruled out explanations in terms of associative learning, unintentional, and olfactory cues. Altogether, the group of 16 dogs outperformed non-human primates in previous studies that applied the same tasks, which led the authors to conclude dogs have at least some elements of a functional theory of mind in their interactions with humans (Maginnity and Grace 2014).

**Fig. 1:**
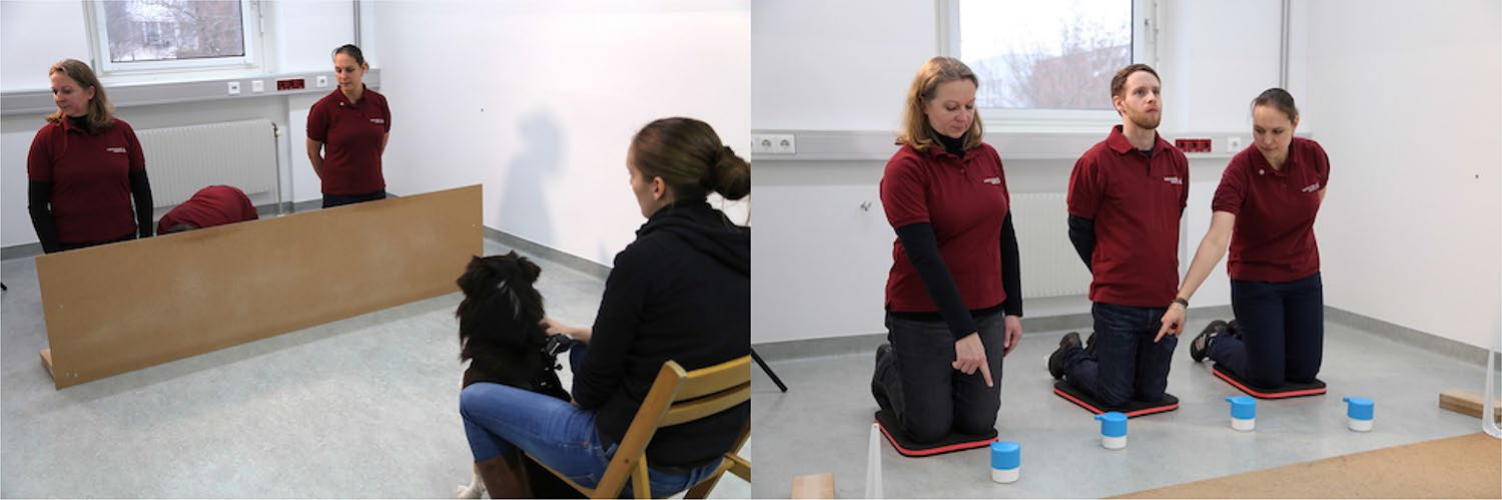
Snapshots of the study testing perspective-taking in dogs (Catala et al. 2017): left: hiding process; right: pointing of the two informants

Although this study confirmed that dogs have a remarkable sensitivity to cues relating to humans’ attentional state, in this case, the visibility of the humans’ eyes and their gaze directions, it remained an open question whether the dogs’ assessment of a human’s knowledge would go beyond *directly observable differences* between the two informants. Therefore, after first replicating the main results of the previous study (Maginnity and Grace 2014), Catala et al. (2017) conducted a variant of the ‘*Guesser–Knower’* task in which the human informants behaved *identically*. Both informants looked in the same direction (actually at a marker on the wall on their right side), but one knelt on the left and one on the right side of the centrally positioned hider (see Fig. 1). Therefore, while exhibiting the same looking behaviour, they differed in whether they could see the baiting process. Importantly, the object of interest to the human (the hiding location) was not visible to the dogs; therefore, they could not simply use the eye-object line (Heyes 1994; Udell and Wynne 2011), but had to infer from the humans’ positions what they could potentially see or not. In other words, they needed geometrical gaze-following (Met et al. 2014) and a basic understanding that seeing leads to knowing. The roles of the informants, the baited containers, and the pointing positions were counterbalanced and pseudo-randomly determined for each trial prior to the tests. From the first trial on, dogs preferred to follow the knowledgeable over the ignorant informant. Obviously, the dogs were able to mentally reposition themselves to imagine what the humans have seen or could potentially have seen. This is not a trivial achievement, because in many non-human species, there is no or little evidence of using the gaze direction of a human experimenter, or a conspecific, as a cue to find hidden food (Fitch et al. 2010; Schloegl et al. 2008).

**Fig. 2:**
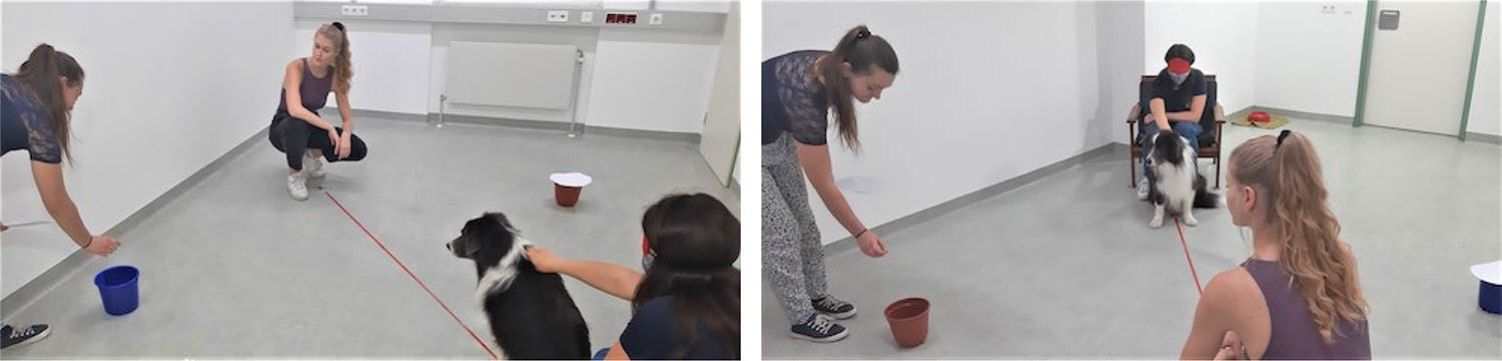
Snapshots of the study testing false belief understanding in dogs (Lonardo et al. 2021): left: the hider is baiting container A; right: the hider has taken the food out from container A and is now baiting container B

## Sensitivity to others’ beliefs

Taking the perspective of another individual is considered a crucial element of theory of mind, but this ability is not exhausted by the capacity to discern what another individual can and cannot perceive from her own point of view. Beyond this, Level 1 perspective-taking capacity is the more advanced capacity to form a mental representation of the visual knowledge of the other (Flavell et al. 1981, 1978). This Level 2 perspective-taking is achieved when an individual understands not only *what* can and cannot be seen from a certain point of view but also *how* a given object is seen or presented (Meunier 2017). The distinction has been confirmed by developmental psychologists who showed that in human infants, Level 1 perspective-taking develops towards the end of the first year of life (Luo and Baillargeon 2007), but Level 2 only 2 years later at the earliest (Moll and Meltzoff 2011). Only then they understand that the same object might appear differently from another’s perspective.

In the human literature, the benchmark test for the understanding of what others have seen in the past and know in the present (“what they believe”) is the ‘*false belief*’ task (Baron-Cohen et al. 1985; Wimmer and Perner 1983). Here, one must take another’s perspective, and not attribute one’s own knowledge to them but have the capacity to recognise and understand someone else’s point of view, i.e., understand that another individual can hold a mistaken perspective. Due to its proposed connection to language and propositional statements, it has been for a long time assumed that theory of mind is a defining (and unique) feature of human cognition (Davidson 1982). Only we humans have the ability to use concepts of intentional mental states, such as intentions, goals, and beliefs, to predict and interpret the behaviour of others. Nonhuman primates are unable to understand or participate in conversation about mental states (Heyes 2003; Penn and Povinelli 2007). The strong connection to linguistic competences was not surprising as traditionally studies have used elicited-response tasks in which children are asked a direct question about an agent’s false belief (Wimmer and Perner 1983). The correct answer to such questions requires the consideration of the information available to agents when interpreting and predicting their actions—even if this information is inaccurate and incompatible with one’s own. Only at about 4 years of age are human children successful in this task of attributing false beliefs to others (Perner 1991). Noteworthily, the ability to understand others’ false beliefs (i.e., reality-incongruent mental states) has been considered a crucial test of theory of mind, because it generates unique predictions of others’ behaviour. Such predictions are impossible solely from the actual states of the world (Dennett 1978).

Great apes seem to understand that their own perspective can differ from reality (Karg et al. 2014; Krachun et al. 2009), but in contrast to anecdotal evidence in favour of chimpanzees' ability to use deception to create a false belief in others, experiments have found that chimpanzees’ deceptive skills have limited flexibility. Perhaps, they deceive and hide, because they have learned rules about the relation of others’ line of sight and their behaviour in the past, rather than understanding the other’s false belief (Heyes 1998). Indeed, until a few years ago, chimpanzees had always failed in tasks that explicitly tested false belief understanding and, in contrast to 6-year-old children, also failed to discriminate their own true perspective with the mistaken perspective of a competitor (Karg et al. 2016). Therefore, the evidence-based conclusion at that time was that "‘putting themselves in the mental shoes’ of others that are really different seems to be a cognitive challenge for them" (Karg et al. 2016, p. 563).

As long as 2 decades ago, developmental psychologists using tasks in which children’s understanding of an agent’s false belief is inferred from behaviours they spontaneously produce revealed that this ability emerges much earlier in human infants. Such spontaneous-response tasks have shown that infants can attribute to an agent a false belief about an object’s location, a false perception of an object, and a false belief about an object’s identity (for reviews, see Baillargeon et al. 2016, 2010; Wellman 2018). This methodological paradigm shift from explicit to implicit false belief tasks was then replicated in investigations of false belief understanding in non-human animals. Using eye-tracking technology to determine where subjects look, primatologists have found that great apes (Kano et al. 2017, 2019; Krupenye et al. 2016) and Japanese macaques (Hayashi et al. 2020) visually anticipate that an actor will search for an object where s/he falsely believes it to be. Despite some concerns about the reproducibility of similar paradigms in human infants, this primate evidence appears to be robust, not least because of similar findings from subsequent behavioural false belief tasks (see below).

The split into implicit and explicit mind-reading tasks has been important for both methodological and conceptual reasons; it opened the door for non-verbal subjects to show some understanding to anticipate what others will do based on their knowledge or belief about the current state of affairs, and it suggests that two different mechanisms may underlie this competence. Mindreading may derive from an innate, domain-specific implicit ability, and a learned, domain-general explicit one (Apperly et al. 2009). This implicit theory-of-mind system is said to be fast, effortless, and automatic (Clements and Perner 1994), and is shared with our nearest primate relatives. The explicit theory-of-mind system develops gradually via domain-general learning in response to social and linguistic input, and is slow, effortful, and heavily reliant upon executive resources, such as working memory (Perner and Lang 1999). It only emerges after children’s fourth birthday and might not be shared with other species (but see Buttelmann et al. 2017). Typically, in explicit adult theory of mind reasoning the content is propositional, i.e., sentence-like, based on a linguistic system for describing different mental states and structuring their content (Astington and Baird 2005).

So, what about dogs? Their performance in the Guesser–Knower tasks is suggestive of Level 1 perspective-taking. But would they also understand that another individual can hold a mistaken perspective? Concerning the latter, only a few years ago, the consensus among canine researchers was that dogs are very skilful at solving social problems, but that they do not understand humans’ knowledge about past events and beliefs (Bräuer 2014). However, the limited number of studies on which this conclusion was based and the surprisingly good performance in the Guesser–Knower tasks provoked a new, intensified attempt. And some evidence for tactical deception in dogs—they distinguished between a cooperative and a competitive human partner, which suggests that they used false signals to modify the behaviour of the receiver (Heberlein et al. 2017a, b)—justifies an optimistic attitude in this respect.

## Dogs react differently to a true and a false belief of human informants

Our approach was to adapt interactive false belief tasks that had been previously employed with human infants (Buttelmann et al. 2009) and apes (Buttelmann et al. 2017; Call and Tomasello 1999). Specifically, the task was to help a human experimenter achieve his goal of opening a box. Subjects watched as an object was switched from one box to another, while the experimenter either witnessed the switch (true belief condition) or not (false belief condition). In both conditions, the experimenter then attempted unsuccessfully to open the box the toy had originally been in. Then, the subjects were asked to help. If they understood what the goal was (opening the box with the object) and also took into account what the experimenter believed (i.e., whether or not he falsely believed there was an object inside a box), they should help open different boxes for the experimenter in the two conditions: the empty box in the true belief condition and the box with the object in the false believe condition. In the true belief condition, they should understand that the experimenter's attempt to get into the first (now empty) box could not be to extract the toy. In contrast, in the false belief condition, if the subjects understand the experimenter’s false belief and wanted to help, they should infer that he wanted the object he thought was in there, and therefore should not simply go to help him open the first box. Instead, they should go to the other box (where the object actually is) and extract the toy for him. While children behaved in this way in both conditions (false belief: 18 out of 25 18-months-old and 20 out of 25 16-months-old; true belief: 21 out of 25 18-months-old but only 14 out of 25 16-months-old) (Buttelmann et al. 2009), the great apes (chimpanzees, bonobos, and orangutans) were above chance only in the false belief condition (Buttelmann et al. 2017).

Cooperation with humans, rather than competition, seemed to us also the most appropriate and natural situation for pet dogs, for example, when reading human communicative intentions and engaging with humans in social games like hiding-finding interactions (i.e., Topál et al. 2009a). In a pre-registered ‘*change of location*’ test with 260 dogs, subjects could retrieve food from one of two opaque buckets after witnessing a misleading suggestion by a human informant (the "communicator") who held either a true or a false belief about the location of food (between-subjects design) (Lonardo et al. 2021). Importantly, this task differs from explicit tests of false belief understanding in that the subject is not asked to predict what the communicator will do on the basis of her mental state. Rather, the experimenters measured whether dogs’ choice of which container to inspect first was (implicitly) influenced by the communicator’s superfluous suggestion. Because this suggestion was identically misleading in both groups, however, a difference in dogs’ choices between groups might reflect that the dogs were sensitive to whether the communicator had perceptual access to the food displacement or not and possibly to the communicator’s subsequent belief.

Crucially, before the tests, dogs were familiarized with the set-up, the experimenters and the possibility of retrieving food from one of the buckets. In particular, dogs were accustomed to the fact that one experimenter (the hider) always hid food in one container first (container A) and sometimes relocated the food to a second container (B) before leaving the room. They also experienced another experimenter (the communicator) suggesting to the dogs where to look for food when they themselves had not witnessed the hiding procedure. This means that during the familiarization, the communicator always suggested to the dog the correct location of food, and thus, the dog could establish a certain degree of trust in this informant.

In the (single) test trial, the dogs’ own witnessing of the events conflicted with the suggestion they received from the communicator about the location of food. Dogs witnessed the initial hiding of food in bucket A and the subsequent displacement into bucket B (see Fig. 2). The transfer from A to B was made obvious by the hider, by showing the food in her hand when walking from A to B. Thus, when the hider then left the room, the dogs knew that the piece of sausage was in bucket B. However, they not only witnessed the hider's actions, they also could see what the communicator, who was located on the opposite side of the room, witnessed. In both experimental groups, the communicator witnessed the hiding of food in bucket A. However, for half of the dogs, food was visibly transferred from container A to container B in the presence of the communicator (true belief condition, TB), for the other half in her absence (false belief condition, FB). Therefore, for half of the dogs, their knowledge about the final location of food was in agreement with the knowledge of the communicator (TB group), and for half in conflict with it (FB group). Crucially, all dogs received a misleading suggestion from the communicator, highlighting with multi-modal signals (including gaze alternation and talking) the wrong container A. The rationale for this was the comparison of two groups of dogs with the same own knowledge of food and the same cueing by the human informant, with the only difference being the informant's knowledge and communicated belief where the food was. Therefore, the main question was whether the two groups of dogs would react differently in response to the same misleading suggestion.

Unsurprisingly, the majority of dogs were not fooled by the human and went straight to the baited (correct) container B. But among those dogs that followed the human informant’s misleading suggestion to go to the empty container A, more did so when the informant had a false belief, i.e., was absent *during* the displacement, than when she had a true belief, i.e., was absent *before,* and therefore present during, the displacement. In a second experiment that controlled for retroactive interference—the disruption of memory of a previously encoded event when this is followed by a second salient event, here the return of the communicator—the communicator in the true belief condition was absent *after* the hider's displacement. However, this manipulation did not change the dogs' performance. Therefore, it is safe to assume that the dogs responded to the communicator's apparent *knowledge* about where food is, which differed according to the *timing* of her absence (whether she was absent or present during the displacement).

Although their differential reaction to the experimental manipulation suggests that dogs take human belief states into account, dogs behaved in an opposite way to human infants and apes in similar paradigms (Buttelmann et al. 2017, 2009; Mascaro and Kovács 2022), and this fact provokes further questions. Have the dogs interpret the two communicators' suggestions differently by attributing to them not just different beliefs but also different intentions? It is a fascinating, but so far highly speculative possibility that dogs in the true belief group interpreted the communicator’s misleading behaviour as deceitful, because during the familiarization trials, this person had proved to be a reliable helper for the dogs but suddenly, in the test, suggested the wrong container. Note that in Buttelmann et al.'s (2017, 2009) helping studies with great apes and infants, the experimenter’s goal was not to communicate to participants the location of the hidden object; therefore, it is unlikely that participants viewed the experimenter as untrustworthy. In contrast, the dogs in the false belief group may have followed the informant’s wrong suggestion, because they attributed to her a false belief and consequently a "justified" mistake in "good faith".

It is important to note that the reversed frequency of following the false belief communicator more likely than the true belief communicator is not the whole picture. There is one noteworthy deviation in the data, which concerns dogs from FCI (Fédération Cynologique Internationale) group 3, terriers. Like other dogs, most followed their own knowledge about the final location of food. Those that did not, however, behaved much like children and great apes; only a few followed the human informant with the false belief, significantly more the human informant with the true belief. Because the sample of terriers tested in Experiment 1 was quite small (*n* = 10), we ran a follow-up experiment in which we tested another group of 40 terriers and—in comparison—40 Border collies. The results confirmed the earlier findings; while only 20% of the terriers in the FB group chose the empty container A, 50% did so in the TB group. This contrast was reversed in the Border collies (55 versus 30%, respectively). Of course, only further tests with even larger samples of pure-breed dogs can clarify these initial and un-predicted data. Meanwhile, we can only speculate about the reasons and seek to find answers from other studies that found breed differences. Unfortunately, these studies concern specific temperament traits (Scott and Fuller 1965; Serpell and Hsu 2005), behaviours (for instance, Kolm et al. 2020), and interspecific communicative abilities such as the tendency to look at a human’s face (Jakovcevic et al. 2010) and to follow pointing gestures (Gácsi et al. 2009), but much less cognitive skills (Gnanadesikan et al. 2020). A possible answer may be found in the working history of breeds, in particular in their cooperative attitudes towards humans. While some breeds have supposedly been selected for cooperating while keeping continuous visual contact with their human partner, others have been selected for working without any human visual contact (Gácsi et al. 2009). Unsurprisingly, Gácsi et al. (2009) found that cooperative workers (e.g., shepherds and gundogs) were more willing than independent ones (e.g., terriers, hounds, greyhounds, and sledge dogs) to follow human distal, temporary pointing gestures. In contrast, when forbidden to eat food, ‘independent workers’ were more skilled at taking their owner’s perspective than cooperative workers (Heberlein et al. 2017a, b). This finding might explain why terriers, which are considered independent breeds, were much less inclined to follow the suggestion of the false belief communicator than Border collies, which are considered cooperative workers (Lonardo et al. 2021).

Irrespective of how the dogs interpreted the communicator's (wrong) suggestion, we would argue that the dogs responded to the different timing of the communicators' absence, which is a much more indirect cue than open eyes, line of sight, illumination, and transparency of barriers. What remains to be clarified is whether this timing difference would serve as an observable regularity in the world that makes the attribution of mental states like knowledge and beliefs unnecessary. Rather than using humans' mental states as mediating elements between their earlier and later behaviour in the false belief task, the dogs may have relied on some innate or learned rule that captures this observable regularity directly (Halina 2018). The most obvious one is this: people look for things where they last saw them (Andrews 2018). Furthermore, even if dogs attributed a mental state to the communicator, our experiment cannot distinguish between the potential attribution of false belief or ignorance (Baillargeon et al. 2010), and hence, the results could be interpreted in terms of Level 1 perspective-taking.

## Conclusion

The recent findings from Guesser–Knower (Catala et al. 2017; Maginnity and Grace 2014) and false belief studies (Lonardo et al. 2021) support the opinion that dogs are exquisite readers of our behaviour (e.g., Udell and Wynne 2011), are capable of Level 1 perspective-taking ability (Kaminski et al. 2011), and might have a rudimentary theory of mind (Horowitz 2011). We, therefore, suggest that the evidence currently favours the hypothesis that (pet) dogs know a lot about seeing and hearing (of humans). Whether this knowledge or inference involves elements of mind-reading remains an open question, however (Buckner 2014; Call and Tomasello 2008; Heyes 1998; Lurz 2011; Penn and Povinelli 2007).

It is conceivable that in most cases in their everyday life, the use of directly observable cues is sufficient, and special conditions, like tasks designed by human experimenters, are necessary to bring their more advanced "human reading" competences—the “elusive cognitive ghost” (Miklósi and Szabó 2012)—to the surface. It is also very likely that such abilities arise from a years-long and intensive cohabitation with humans, through socialization with humans and experience with the stimuli employed in the tasks used to test it in the laboratory (Benz-Schwarzburg et al. 2020; Udell et al. 2011; Udell and Wynne, 2008, 2011; Wynne 2016). Dogs' potential to interpret and predict human behaviour, perhaps using rules like "they look where they have seen it last", would then not be based on an innate module, but would arise in the course of development through the experience of the dog’s own behaviour and that of humans (Roberts and Macpherson 2011). Specifically, in addition to the phylogenetic changes (*inflection*), in which natural selection biases the *input* to a cognitive mechanism (Heyes 2003), here the signals from humans, the human-reading ability may develop as a kind of *ontogenetic construction*. As in human children, this developmental process would generate adaptive change to the cognitive mechanism, that is, to its rules and/or representations (Heyes 2003). Indeed, when tested in the false belief task, older dogs were significantly less likely to choose the empty bucket (A) that was suggested by the human communicator, irrespective of condition (Lonardo et al. 2021).

Maybe, they have adapted their behavioural rules over the years, and learned not to trust humans blindly, but hesitate before following their suggestions. At the moment we do not know if dogs can acquire higher-level understanding of perspectives, such as an understanding that the same object might appear differently from another perspective (Level 2). However, what we can say is that, due to their success in most perspective-taking paradigms, even when behavioural differences are made ever more subtle between conditions, dogs are a promising species to test the hypothesis that non-human animals might possess theory-of-mind abilities. This would require being able to infer from observable behaviour unobservable mental states and being able to understand that those mental states are the cause of subsequent behaviour (Heyes 1998). This hypothesis needs to be tested with experiments that involve identical behaviours of the experimenters (but differing mental states) between conditions, and rule out alternative explanations that have already been addressed in the primate literature (Krupenye and Call 2019). The door is now wide open to consider testing dogs in more challenging situations and novel experimental paradigms. For instance, can dogs use their own past experience to infer what a competitor can see?

## Data Availability

No data was used for the research described in the article.
